# Engineering a pH responsive pore forming protein

**DOI:** 10.1038/srep42231

**Published:** 2017-02-08

**Authors:** Matic Kisovec, Saša Rezelj, Primož Knap, Miša Mojca Cajnko, Simon Caserman, Ajda Flašker, Nada Žnidaršič, Matej Repič, Janez Mavri, Yi Ruan, Simon Scheuring, Marjetka Podobnik, Gregor Anderluh

**Affiliations:** 1 Department of Molecular Biology and Nanobiotechnology, National Institute of Chemistry, Hajdrihova 19, 1000 Ljubljana, Slovenia; 2 Department of Biology, Biotechnical Faculty, University of Ljubljana, Jamnikarjeva 101, 1000 Ljubljana, Slovenia; 3 Department of Computational Biochemistry and Drug Design, National Institute of Chemistry, Hajdrihova 19, 1000 Ljubljana, Slovenia; 4 U1006 Institut National de la Santé et de la Recherche Médicale (INSERM), Université Aix-Marseille, Parc Scientifique et Technologique de Luminy, 163 avenue de Luminy, 13009, Marseille, France

## Abstract

Listeriolysin O (LLO) is a cytolysin capable of forming pores in cholesterol-rich lipid membranes of host cells. It is conveniently suited for engineering a pH-governed responsiveness, due to a pH sensor identified in its structure that was shown before to affect its stability. Here we introduced a new level of control of its hemolytic activity by making a variant with hemolytic activity that was pH-dependent. Based on detailed structural analysis coupled with molecular dynamics and mutational analysis, we found that the bulky side chain of Tyr406 allosterically affects the pH sensor. Molecular dynamics simulation further suggested which other amino acid residues may also allosterically influence the pH-sensor. LLO was engineered to the point where it can, in a pH-regulated manner, perforate artificial and cellular membranes. The single mutant Tyr406Ala bound to membranes and oligomerized similarly to the wild-type LLO, however, the final membrane insertion step was pH-affected by the introduced mutation. We show that the mutant toxin can be activated at the surface of artificial membranes or living cells by a single wash with slightly acidic pH buffer. Y406A mutant has a high potential in development of novel nanobiotechnological applications such as controlled release of substances or as a sensor of environmental pH.

Protein engineering is a well-established research field with many examples of successfully engineered proteins that are used in industry or medicine^1^. Most of the work has been done on enzymes due to their commercial potential, although other proteins such as antibodies have also been successfully modified^2^. Pore-forming proteins (PFPs) are a heterogeneous group of proteins with an ability to efficiently form pores in lipid membranes^3^,^4^. Natural PFPs are used for attack or defense by many organisms and they are abundant in bacteria, where they serve as virulence agents. Pores formed by PFPs are of nanometer dimensions (hence the name nanopores) and can be extremely stable and resistant to high temperatures and detergents. Some of the nanopores have been recently successfully engineered^5^ and employed in various sensing applications^6^,^7^,^8^,^9^, with exceptional successes in DNA sequencing^10^, and used for other purposes, e.g. semisynthetic nanoreactors for studying covalent chemistry at the single-molecule level^11^. There is a growing need to engineer and modify PFPs in order to generate nano-pores with better properties for sensing or nanopores that are responsive to physical or chemical factors from the environment. Here we engineered a PFP in order to be able to control its activity by pH.

Listeriolysin O (LLO) is a member of cholesterol-dependent cytolysins (CDC), a family of PFPs that selectively perforate cholesterol-rich lipid membranes^4^. It is a major virulence factor of the bacterium *Listeria monocytogenes*, a food-borne intracellular pathogen that leads to a high mortality rate in humans despite available antibiotic treatments^12^. LLO has a crucial role in cellular invasion of *L. monocytogenes*, where LLO-mediated membrane damage, together with other factors, allows bacterial escape to the cytosol of the host cell and consequent replication of bacteria and spread to other cells and tissues^13^,^14^,^15^,^16^. LLO is a 56 kDa rod-shaped protein composed of four domains (D1-D4, Fig. 1) and produced as a soluble monomer^17^ and presumably follows the proposed CDC molecular mechanism of pore formation^18^. It efficiently binds to cholesterol rich lipid membranes^19^ and oligomerizes into arc-like structures on the surface of the membranes^20^,^21^. In the next step helix bundles 1 and 2 (HB1 and HB2, Fig. 1) from domain 3 reorganize into β-hairpins that finally traverse the membrane and become transmembrane hairpins. This is accompanied by rearrangement of other domains, which brings the molecule closer to the membrane and results in the reduction of height of the final inserted LLO molecule^20^. Inserted LLO arcs are conductive pores and are able to interact between themselves to form larger supramolecular assemblies, which can completely destroy the lipid membrane over longer time scales^20^,^21^. LLO is especially interesting for engineering because of its naturally present pH dependent stability, which is important for its biological role. At physiological pH 7.4, found in the cytosol and extracellular environments, combined with temperatures above 30 °C it rapidly and irreversibly aggregates to a non-functional form, while its thermal stability is significantly higher at a lower pH of ~5.5 found in late endosomes^22^,^23^.

LLO has been used as a tool in medical applications, such as vaccine adjuvans, or gene and other cargo delivery agents^24^,^25^,^26^,^27^ and there is clearly a high demand for understanding its biochemical and biophysical properties. Here we engineered an LLO mutant Y406A, which has an unprecedented pH-dependent pore-forming ability mediated through allosteric effects. The Y406A mutant retained the LLO capacity to bind to lipid membranes and oligomerize, however, the membrane insertion step was crucially dependent on pH. It was possible to activate the membrane-bound mutant by lowering the pH, as shown in model membranes and in a cellular setup. LLO thus represents an example of PFP that can be engineered to allow controlled cellular membrane permeabilization by external cues such as pH.

## Results and Discussion

**Figure 1:**
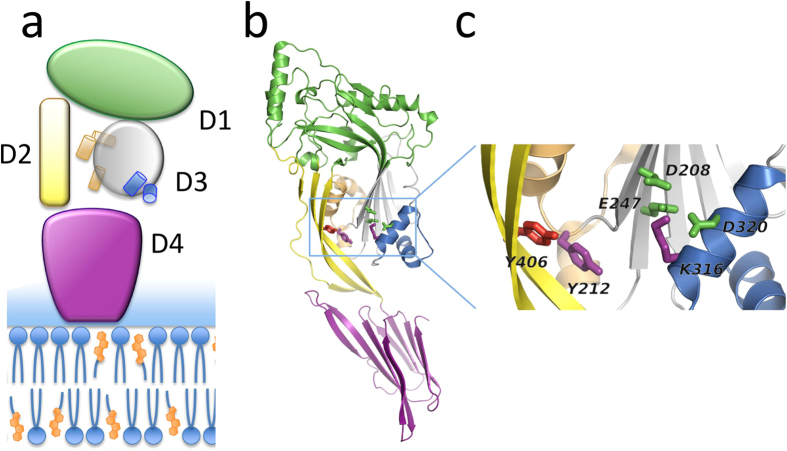
Domain organization in LLO. (**a**) Schematic drawing of LLO bound to the lipid membrane. The protein and the membrane are drawn to scale. LLO associates with the membrane with D4 (magenta), D2 (yellow) connects D4 to the pore-forming D1 (green) and D3 (grey) domains. D3 contains the HB1 (light brown cylinders) and HB2 (blue cylinders) that unwind and form transmembrane hairpins during pore formation. Majority of CDCs require relatively high concentrations of cholesterol (orange) in the membranes. (**b**) Crystal structure of LLO (PDB ID: 4CDB) in ribbon representation, the residues mutated in this study are in the central part of the molecule (in D2 and D3) and depicted as sticks. (**c**) Close up of D2-D3 interface in (**b**). The pH sensor (acidic triad D208, E247, D320; green sticks) is located in D3, between the central β-sheet and HB2.

LLO stability is dependent on pH and temperature^22^,^23^,^28^. A pH-sensor composed of three negatively charged amino acids (D208, E247, D320) was identified in D3^22^ (Fig. 1). At pH values above 6 and temperatures above 30 °C, the HB2 of D3 unfolds due to charge repulsion of amino acids in the sensor. The unfolding leads to premature aggregation of the protein in solution in the absence of the membrane, and causes its inactivation^22^,^23^. LLO permeabilizing potency is, however, only marginally affected by pH when protein is assayed at temperatures below 30 °C^19^,^23^. Based on the LLO structure (PDB ID: 4CDB, ref. 17.) we reasoned that we could engineer an LLO variant with significantly different permeabilizing activity at different pH values and room temperature by affecting the immediate environment of the pH-sensor. D320 of the sensor is located in one of the helices in the HB2 region that unfold into transmembrane β-hairpins during the pore-forming mechanism and participate in formation of the final β-barrel pore. On the same α-helix we noted a positively charged amino acid K316. Its side chain faces towards the center of the pH-sensor and may thus neutralize the negative charge of the pH sensor at high pH, stabilize the HB2 in the α-helical arrangement, and thus affect permeabilizing activity (Fig. 1c). Both D320 and K316 are located close to the interface of D2 and D3. Hydrogen bonds and salt bridges are present along the entire interface between D2 and D3^17^,^29^. Weak contacts between the domains are necessary to accommodate conformational changes that occur during pore formation, namely the proposed bending of D2^29^,^30^. The aromatic residue Y406 is located at the interface between D2 and D3 (Fig. 1c) and contributes to the stability of this interface through ionic and hydrophilic interactions^17^, and possibly also through hydrophobic or π-π interactions with Y212 (Fig. 1c). For efficient membrane permeabilization D2-D3 interface has to be disrupted. Therefore, we reasoned that the replacement of Y406 side chain with a smaller residue (like Ala) could affect the strength of interactions, weakening the interactions between D2 and D3 and allosterically affect the environment of K316 and the pH-sensor.

**Figure 2:**
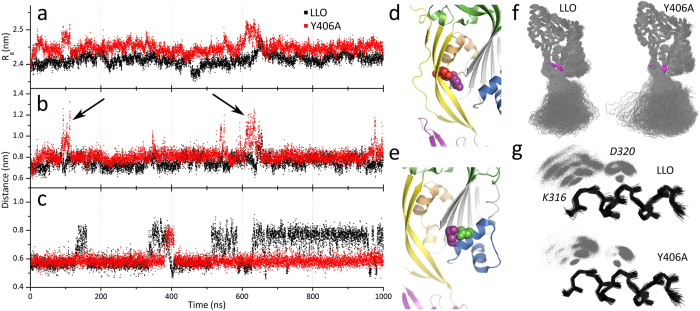
Equilibrium MD simulations of LLO and Y406A. (**a**) Y406A mutant is more flexible than LLO, which is evident from radius of gyration (R_g_) of domains D1-D3. Domain D4 is excluded from calculations of R_g_ to prevent the interference of highly mobile D4 in both variants^30^. (**b**) The distance between Cα atoms of residues A/Y406 and Y212 (entire residues shown as red and magenta spheres, respectively, in panel (d)). Two major peaks (black arrows) at ~100 ns and ~630 ns correspond to partial opening of D2-D3 interface. (**c**) The distance between K316 and D320 centers of mass for LLO and Y406A is 0.66 ± 0.1 nm and 0.59 ± 0.04 nm, respectively. (**d**) Residues A/Y406 as red spheres and Y212 as magenta spheres in the structure of LLO. (**e**) Residues K316 as magenta spheres and D320 as green spheres in the structure of LLO (**f**) Aligned backbone traces of LLO and Y406A mutant (grey). D1 was used to align the snapshots from the MD simulation. Residues 406 and 212 are visible as magenta spheres. (**g**) Dynamics of K316 and D320 side chains. Backbone trace of one α-helix from HB2 is shown as black lines. Side chain atoms of K316 and D320 are shown as grey dots.

We first tested this idea by molecular dynamics (MD) simulations of both LLO and Y406A mutant. MD simulations showed that the radius of gyration of D1-D3 domains is larger for Y406A mutant compared to the LLO throughout the simulation (Fig. 2a). The striking difference between LLO and Y406A is in the D2-D3 interface mobility, where the distance between Cα atom of residue 406 in D2 and Cα atom of residue Y212 in D3 drastically increases two times during the 1 μs long simulation in the mutant (Fig. 2b, Supplementary Fig. 1A), indicating the opening of the interface between D2 and D3. This displacement event is largely the combination of D2 straightening and HB2 displacement from its original position (not shown). Another interesting feature of the Y406A mutant is the changed flexibility of the residue K316 that is located on the HB2 region (Fig. 2c,e,g). We observed significantly less fluctuation in D320-K316 distance (Fig. 2c), which is juxtaposed to the pH-sensor, in comparison to the wild-type protein. In LLO this distance between centers of masses ranges between ~0.7 nm to ~1 nm, while in Y406A the distance is fixed and close to 0.7 nm during the simulation. This suggests a stronger interaction of the basic K316 side chain with the acidic side chains in the pH-sensor of the mutant. pKa calculations showed that higher flexibility of the D2-D3 interface and allosteric effects the Y406A mutation may influence the pKa of multiple amino acid residues in D3 (Supplementary Fig. 1B). Most notable is the E247 with a decrease of the calculated pKa from 5.2 ± 2.5 to 3.7 ± 1.1. This suggests that the glutamate is less likely to be protonated in Y406A at higher pH values and thus more likely to form interactions with neighboring residues and Na^+^ ions, which would represent a barrier for HB2 unfurling into the transmembrane β-hairpin. The Y406A mutation seems to push the equilibrium of E247 toward deprotonation in contrast to the LLO, where there is more fluctuation between the two states (Fig. 2g). This could lead to a pH-dependent pore-forming activity of the Y406A mutant, with lower pH increasing protonation level of negatively charged amino acid side chains thus decreasing the charge and enhancing detachment of HB2 from D3.

The MD simulation results altogether indicate that the Y406A mutant adopts a conformation with larger interdomain flexibility, which influences also the mobility of side chains in and around the pH-sensor, making them more rigid at higher pH values. To check whether these structural changes also affect thermal stability of Y406A, we performed differential scanning fluorimetry. Indeed, stability of Y406A was lower by about 4 °C compared to the wild-type LLO (Supplementary Fig. 2). Both proteins still share a very similar pH dependence profile, which shows that both are most stable at pH ~5.5. Aggregation studies further confirmed these results (Supplementary Fig. 2).

**Figure 3:**
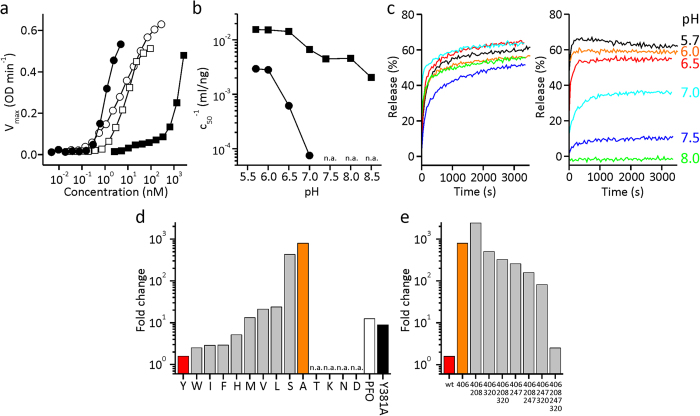
Permeabilization properties of the LLO Y406A mutant. (**a**) Hemolysis maximal rate (OD min^−1^) at different protein concentrations of LLO (solid circles) compared to Y406A (solid squares). PFO (open circles) and a corresponding PFO Y381A mutant (open squares) are shown for comparison. Experiments were performed at pH 7.4 and room temperature. (**b**) Concentration of LLO or Y406A needed to achieve half of the maximal hemolysis rate at different pH values. n.a., not active. Symbols as in 3a. (**c**) Calcein release from LUVs induced by the LLO (left) and Y406A mutant (right). The concentration of proteins was 188 nM. (**d**) Fold change of hemolytic activity of LLO (red) and LLO mutants at position 406 tested at pH 7.4 and 5.7. Higher value represents a more pH-dependent hemolytic behavior. PFO (white) and PFO Y381A mutant (black) are shown for comparison. (**e**) Fold change of hemolytic activity of LLO (red) and a set of LLO mutants. Mutants have 1 to 4 point mutations that are coded with an indication of the mutated residue (Y406A, D208E, E247M, D320K).

To check how the Y406A mutation affected the permeabilizing activity of the mutant protein we first assayed the hemolytic activity of the mutant and compared it to LLO. It is clear that the hemolytic activity of the mutant was almost three orders of magnitude lower in comparison to the LLO (Fig. 3a). This reduction of activity was not observed for the homologous protein perfringolysin O (PFO) from *Clostridium perfringens*. PFO is an archetypal member of CDCs that does not possess a pH-sensor^22^. It, however, contains a tyrosine Y381 at the position equivalent to Y406 and the mutation to alanine does not reduce the hemolytic activity (Fig. 3a). Next we assayed activity of LLO and Y406A at different pH values at room temperature and noticed significant loss of activity in Y406A at pH values higher than 6.5. At lower pH values the Y406A mutant still shows significant hemolytic activity, comparable to the wild-type protein (Fig. 3b). We independently confirmed this behavior of Y406A by a calcein release from large unilamellar vesicles (LUVs) composed of POPC:Chol (1:1; mol:mol) (Fig. 3c). Calcein release from LUVs at room temperature was found not to be dependent on the pH when adding LLO, while Y406A mutant was found to release calcein to a similar extent as the LLO only at pH values below 7.0. Ability of Y406A to release calcein was drastically reduced at pH higher than 7.0 and it was found to be inactive at pH 8.0 (Fig. 3c).

We designed a number of mutants in order to better understand the properties and behavior of Y406A, and tested their hemolytic activity. We expressed the results as the n-fold decrease of hemolytic activity at pH 7.4 in comparison to 5.7. This value is approximately 1.6 for the LLO and ~800 for Y406A (Fig. 3d). We first checked the effect of other amino acid side chains at position 406 and realized that the wild-type behavior is retained by amino acid substitutions that introduce aromatic or bulky hydrophobic side chains (for example Trp, Ile, Phe). Amino acids with charged or polar side chains were not hemolytically active (Lys, Asp, Asn, Thr), while small and uncharged side chain residues seemed to induce the pH-dependent behavior (Ser, Ala) (Fig. 3d). Because Ala or Ser are not under direct influence of the environmental pH we concluded that some other part of the molecule might allosterically respond to the Y406A mutation, as hypothesized and predicted by modeling. These results also reinforced the observation that position 406 is important for interdomain contacts and that the interface between domains 2 and 3 is stabilized, at least in part, by hydrophobic interactions. We then further checked whether the pH-dependent behavior could be affected by mutating amino acids from the sensor to corresponding amino acids found in PFO. Indeed, we observed the gradual reversal to the wild-type LLO phenotype when introducing a single or double mutants, while the triple mutant, in which all three amino acids of the pH sensor were mutated to PFO residues, retained the wild-type’s properties with a ratio of 2.5 (Fig. 3e). These results altogether indicate that we were able to abolish the artificially introduced pH-dependence with three mutations in the pH-sensor in D3 to reflect the state in PFO and highlight the importance of the pH-sensor and the allosteric connection between the distal interdomain region and the pH-sensor.

In summary, the mutation of Tyr406 affected permeabilizing activity in a way that the mutant retained its activity only at low pH-values and this is mediated through effects involving amino acids of the pH-sensor. Obviously, the higher pH values stabilize this part of the molecule upon mutation, through interactions of neighboring amino acids and amino acids from the sensor, in a conformation that does not allow unfolding of HB2 and thus prevent the hemolytic activity. In order to use the Y406A mutant for applications we wished to explore its properties and mechanism of pore formation in comparison to the wild-type protein. Modeling suggested that the observed effects involved only parts of D2 and D3 and no other domains. Therefore, we first checked whether the Y406A mutant retained the membrane-binding capacity mediated by D4 and was able to oligomerize at the surface of lipid membranes similar to the LLO.

We characterized the lipid membrane-binding ability of the LLO and Y406A mutant by several independent assays. Protein binding to membranes was first analyzed by a sedimentation assay, where proteins were added to multilamellar vesicles (MLVs) composed of POPC:Chol and then centrifuged to pellet the proteins bound to MLVs. SDS-PAGE of the pelleted fraction and the supernatant showed that the binding of the Y406A mutant was similar to the LLO (Supplementary Fig. 3A). This experiment also confirmed that pH does not have a major role in binding of LLO or the mutant, if large cholesterol concentrations are present in membranes, as documented in Bavdek *et al*.^19^. The Y406A mutant also retained cholesterol specificity, similar to the LLO, as it only weakly associated with MLVs composed of POPC alone (Supplementary Fig. 3A). Binding of Y406A mutant to a lipid membrane was further assessed by measuring the change in tryptophan fluorescence and surface plasmon resonance analysis (SPR) (Supplementary Fig. 3). An increase in the intrinsic tryptophan fluorescence is an excellent indication of D4 association with lipid membranes, since six out of seven LLO Trp residues are located in domain 4. We observed an increase and blue shift of tryptophan fluorescence in the presence of LUVs for both proteins at acidic and alkaline pH (Supplementary Fig. 3B). The SPR experiments further confirmed robust binding of Y406A to the lipid vesicles that contained high concentrations of cholesterol and that the binding was not dependent on pH (Supplementary Fig. 3C).

**Figure 4:**
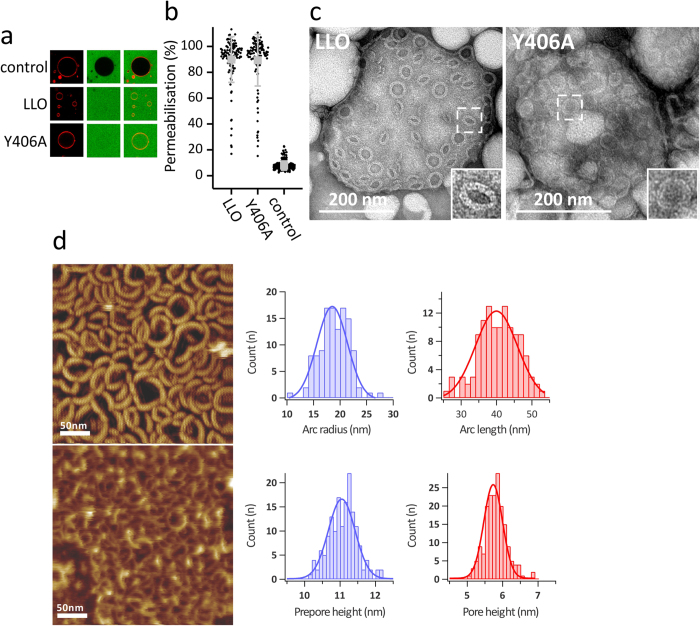
Structural characterization of Y406A pores. (**a**) Permeabilization of GUVs (left, red membrane stain) for fluorescent dextran (middle, green stain) by the LLO and Y406A (the right panels represent the superimposed images). The concentration of proteins was 100 nM in buffer with pH 5.5 and at room temperature. (**b**) Quantitative comparison of permeabilizing activity of LLO and Y406A for fluorescent dextran 70 kDa after 30 min incubation. Control did not contain any protein. An average ± S.D. is shown in grey, together with values for each individual GUV. Each point represents permeabilisation for a single vesicle; ~100 GUVs were analysed for each protein. (**c**) TEM images of multilamellar vesicles composed of POPC:Chol incubated in the presence of the LLO or Y406A at pH 5.5 and at room temperature. Two selected assemblies are enlarged in the inset. (**d**) AFM image of LLO (top) and Y406A (bottom) arcs as seen on POPC:Chol 1:1 lipid membrane at pH 5.6 and room temperature and associated quantification of Y406A arcs.

**Figure 5:**
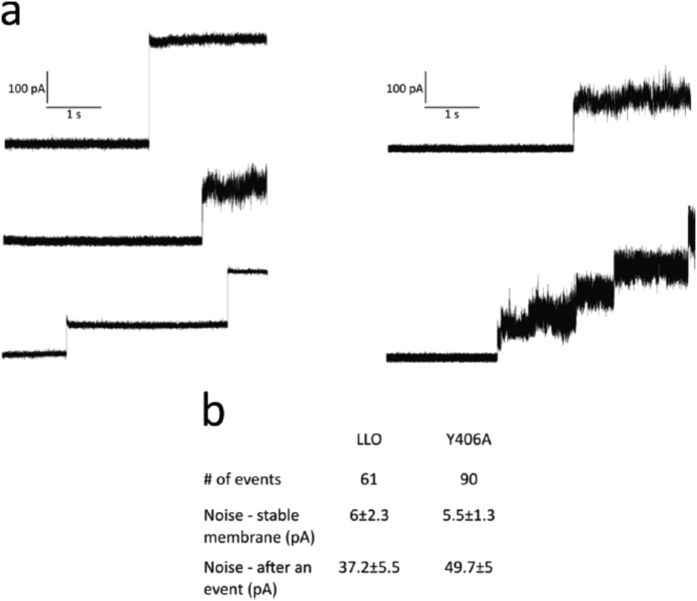
Electrical measurements of LLO and Y406A pores on PLM (DPhPC:POPC:Chol) at pH 5.5. (**a**) Sample current traces of LLO (left column) and Y406A (right column) with one or more pore insertions. (**b**) 16 measurements were performed with each protein and standard deviation (noise) (pA) measured at 5 s intervals.

To determine the properties of pores formed by Y406A we first wanted to explore the size of pores. Using giant unilamellar vesicles (GUVs), large enough to visualize them by confocal microscopy, we monitored pore formation at pH 5.5 by following fluorescent dextran uptake from an external solution to the interior of the GUVs through pores formed in the membrane. We used a 70 kDa dextran, which is ~12 nm in diameter. We could not find significant differences in permeabilization between the LLO and Y406A, indicating that the pore size is similarly large in both proteins (Fig. 4a,b). We also imaged the pores by transmission electron microsopy (TEM) and atomic force microscopy (AFM). TEM revealed that the LLO mostly formed arcs that were clustered together in slit pores^17^ and larger assemblies that looked similar to full circular pores^31^. These assemblies had a clearly resolved and ordered structure of the pore edge (inset in Fig. 4c, left). Y406A formed similar circular aggregates, but were less well structured and defined at the edges (Fig. 4c, right). The circular line is regular and smooth in LLO, while in Y406A the circular line is often ruffled or in some cases seems to be disturbed. This was confirmed by high-speed AFM^32^, which was previously used to image LLO pore formation and dynamics^21^, and other pore forming toxins^33^,^34^. AFM showed arc-shaped structures of Y406A, similar to the LLO, however with some notable differences: the arc-diameter was smaller in comparison to LLO (19 ± 3 vs 29 ± 3 nm^21^; Fig. 4d), while the arc length was similar (40 ± 6 vs 47 ± 6 nm^21^; Fig. 4d). AFM also allowed quantifying the height of membrane-adsorbed and -inserted protein species. LLO monomers that are not yet inserted in the membrane top the membrane by approximately 10.5 nm, while inserted LLO molecules protrude about 7.5 nm in height^20^,^21^. Adsorbed Y406A was also about 10.5 nm in height, however, the extramembraneous part of the inserted protein is lower and protrudes only about 5.5 nm (Fig. 4d). These results altogether confirm that the mutant is able to interact with lipid membranes and that it can oligomerize into structures similar in shape to the LLO. However, there are some structural differences due to the Y406A mutation and the pore seems to have a modified structure compared to the LLO pore. We confirmed this with electrical measurements on planar lipid membranes. We observed the insertion of LLO and Y406A pores into lipid membrane only at pH 5.5, while at pH 7.4 we observed no pore insertion events. As expected the Y406A pore conductance was smaller in comparison to the LLO pore (5.4 ± 0.7 nS vs 7.2 ± 1.2 nS, respectively) and in accordance with previous results^19^,^20^. Interestingly the standard deviation (noise) after the insertion of Y406A pore into the membrane was increased 1.3 fold in comparison with LLO pores (Fig. 5), indicating that Y406A pores are looser assemblies with a higher flexibility compared to LLO pores. We believe that the mechanism of pore formation is similar for both proteins and that only particular steps in this mechanism may be are affected, i.e. insertion into the lipid membrane. This is supported by the AFM data, which show that the height of Y406A pore above the membrane is slightly shorter. On the other hand, the higher flexibility of D2-D3 interface, as seen in MD simulations may affect the interactions of D2 with other protomers within oligomeric assemblies, or the interaction of D2 with either D4 or D3 within the same protomer in the pore state. Altogether, these results support the known plasticity of the LLO molecule in ways it can interact with the lipid membrane and form pores^20^,^21^.

**Figure 6:**
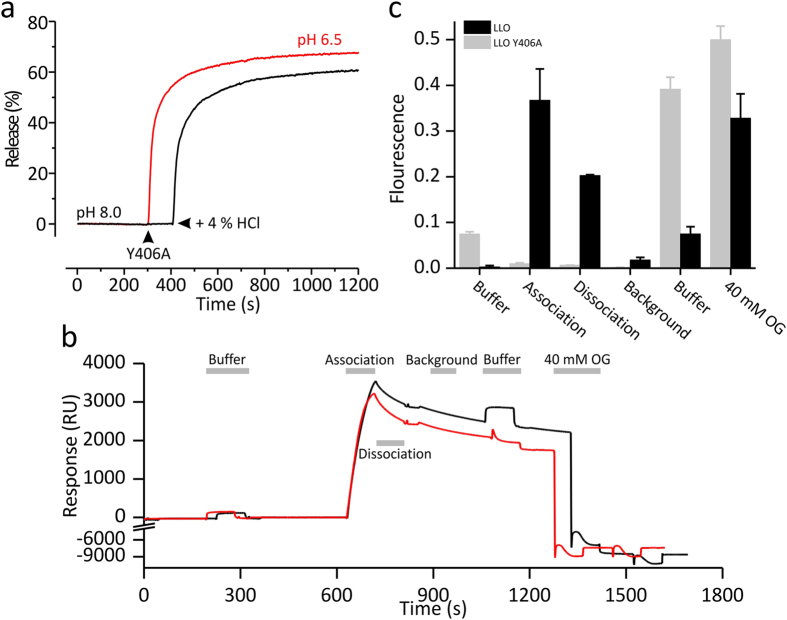
Activation of Y406A on model lipid membranes by slightly acidic pH. (**a**) Calcein release from LUVs. Red and black lines show calcein release at pH 6.5 and 8, respectively. Arrowhead indicates when Y406A was added to LUVs in solution in both cases. Acidification of the environment to pH 6.5 was achieved with addition of small volume of HCl at approximately 400 s (also indicated by an arrowhead). (**b**) SPR sensorgrams of LLO (black line) and Y406A (red line) activation experiment. Grey lines indicate when the solution coming from the flow cells was collected and used for fluorescence measurements. (**c**) Calcein fluorescence in fractions collected during the SPR assay presented in (**b**). Fraction “Buffer” represents a wash with a buffer with a pH of 5.7. 40 mM OG represent a washing step with detergent octyl-glucoside, which removed all remaining material from the surface of the sensor chip (two injections of OG across sensor chip are shown in (**b**), however, all calcein was released only during the first injection, which is presented in the graph).

Altogether these results indicate that Y406A at low pH values can bind to lipid membranes and oligomerize to form pores with similar efficiency and with similar properties as the LLO. The unique features of the mutant present an attractive possibility for its applications, especially in cases where the mutant bound to lipid membranes at physiological or slightly basic pH could be activated by a brief exposure to solutions with a low pH value. We used several different independent approaches to show that this is indeed a special feature of this mutant. We bound the Y406A mutant to calcein-loaded LUVs at pH 8.0 and noted that no calcein was released, in agreement with results presented in Fig. 3c. When the solution was acidified to about pH 6, a rapid calcein release was noted, indicating that the protein was activated and formed pores (Fig. 6a). We also used a previously developed SPR approach to directly monitor binding of proteins to a membrane and release of fluorescent probes upon activation^35^. We immobilised calcein-loaded LUVs composed of POPC:Chol on the surface of the sensor chip and monitored binding of the LLO or Y406A mutant (Fig. 6b). The binding was similar for both proteins, in agreement with other assays (Supplementary Fig. 3). We collected the solution coming from the flow-cell and measured the fluorescence (Fig. 6c). While the mutant clearly associated with LUVs in a similar manner as the wild-type protein, it did not release any calcein during association or dissociation phases (Fig. 6c). The calcein was released only when the buffer with the low pH was injected across the surface of LUVs with bound Y406A, again indicating activation of the protein and formation of pores.

**Figure 7:**
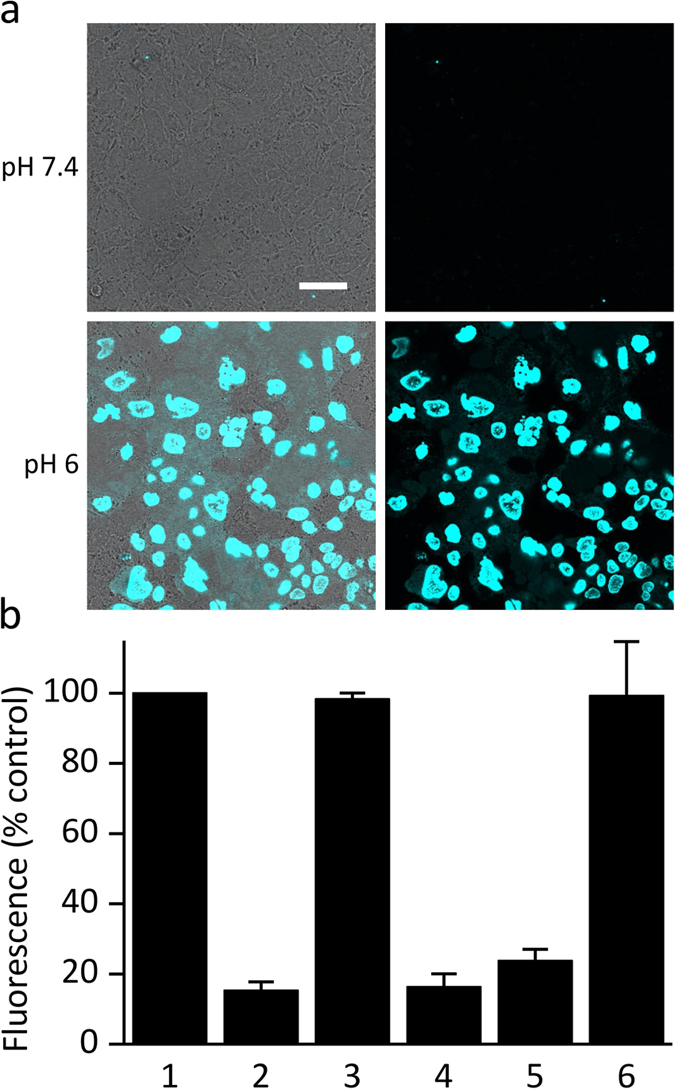
Activation of Y406A on the surface of Caco-2 cells. (**a**) Cells exposed to Y406A and washed with the medium at two different pH values. Left column, differential interference contrast image of Caco-2 monolayer superimposed with fluorescent image; Right column, fluorescence microscopy of the same monolayer. (**b**) Quantification of fluorescence. Lines 1 and 2, positive and negative control, respectively, at pH 7.4; Lines 3 and 4, positive and negative control, respectively, at pH 6; Line 5, Caco-2 cells washed with medium at pH 7.4; Line 6, Caco-2 cells washed with medium at pH 6.

Finally, we checked the possibility of protein activation in a cellular setup. We had previously used Caco-2 intestinal epithelial cells for monitoring pore-forming activity of LLO^36^. The cells were exposed to the mutant at pH 7.4 and after brief incubation washed with media at two different pH-values. When pores formed upon activation at low pH, Sytox green entered the cells and stained the cell nuclei. We noted no fluorescence when Caco-2 cells were washed at pH 7.4, but cells were stained with Sytox green upon washing at pH 6 (Fig. 7).

Usage of protein engineering to achieve activity at a certain pH is a well-known approach to improve or adjust the optimum activity of a certain protein, particularly enzymes. There are examples where non-enzymatic activity or other properties of proteins were designed to respond specifically to changes in the environmental pH. Green fluorescent protein and its related fluorescent proteins have been extensively engineered since they enable an excellent insight into pH variations in living cells^37^,^38^. Other examples are: engineering pH responsive fibronectin domains^39^, pH dependent binding of antibodies^40^,^41^, pH dependent protein switches^42^, pH-dependence of protein A binding^43^ and an example of a pore-forming peptide GALA^44^. Most of these later examples depend on engineering histidine residues, which have pKa values close to the physiological pH or its neighboring residues. Here we designed a pH-responsive pore-forming protein. We achieved a pH-responsive phenotype by mutating an amino acid residue that is not a histidine and is distal from the protein region that mediates the pH-dependent behavior of LLO. Protein engineering by taking advantage of such allosteric effects is a more recent development^45^ that connects the need to affect the activity of proteins without compromising their structural properties. We show here that the mutant is still well-soluble in aqueous solution, is slightly less stable than the wild-type protein due to an important structural role of the residue Y406, however, it maintains its membrane-binding capacity in a wide pH range. In contrast, it expresses a permeabilizing phenotype only at pH-values that are close to the acidic environment of the phagosomal vacuoles. The ability to specifically induce pore formation by sensing environmental pH has potential for applications that we explored here in a cellular setup. Experiments on cell cultures exposed to Y406A showed that it is possible to induce a controlled cell membrane permeabilization by simply washing the cells in a medium with lower pH. LLO has proven to be amenable for engineering and its further developments will contribute to the growing need for protein nanopores used in sensing as well as in cell biology applications.

## Methods

### Materials

All materials in this study were obtained from Sigma-Aldrich, USA unless stated otherwise.

### Mutagenesis

Site-directed mutagenesis was performed as described in Shenoy and Visweswariah^46^. Briefly, a single oligonucleotide containing the desired mutation was used in a PCR reaction followed by the restriction of target methylated DNA. Obtained DNA was transformed into *Escherichia coli* DH5α cells and suitable mutants were confirmed by DNA sequencing of the entire LLO encoding gene.

### Protein expression and purification

Proteins were expressed in *E. coli* BL21(DE3) pLysS cells in Terrific Broth (TB) medium supplemented with phosphate buffer and antibiotics. Cells were grown at 37 °C with shaking until absorbance at 600 nm reached ~1 and induced with 0.5 mM isopropyl 1-thio-β-D-galactopyranoside. After shaking for 20 hours at 20 °C the cells were harvested by centrifugation at 4 °C and 4410 × *g* for 5 minutes. Cells were resuspended in ~10 ml of lysis buffer (50 mM NaH_2_PO_4_/Na_2_HPO_4_, 250 mM NaCl, 10% (v/v) glycerol, pH 6.5) and 2-sulfanylethanol was added to a final concentration of 5 mM. Protease inhibitor phenylmethanesulfonyl fluoride (PMSF) was added to a final concentration of 2 mM. Cell suspension was sonicated and debris were removed by centrifugation at 50 000 × *g*, 4 °C for 1 h. The supernatant was filtered through 0.22 μm filter, aliquoted and frozen at −80 °C until use. For protein purification the cell lysate was loaded to an IMAC (Ni-NTA, Qiagen) column, washed extensively with the wash buffer (50 mM NaH_2_PO_4_/Na_2_HPO_4_, 250 mM NaCl, 60 mM imidazole, pH 6.5) and the bound fraction eluted with the same wash buffer except the imidazole concentration was 300 mM. TEV protease was added to a final concentration of ~30 μg/ml and the solution was dialyzed 1:60 overnight at 4 °C in a dialysis buffer (30 mM NaH_2_PO_4_/Na_2_HPO_4_, 150 mM NaCl, pH 6.5). Flow-through after reverse mode IMAC containing purified protein was concentrated with Amicon Ultra 30 kDa MWCO (Merck, Germany) to several mgs/ml. Final step was size exclusion chromatography with Superdex 200 (GE Healthcare, UK) which was first washed with GF buffer (20 mM 2-(N-morpholino)ethanesulfonic acid (MES), 150 mM NaCl, pH 5.7). Fractions of pure protein (determined with SDS-PAGE) were pooled and concentrated to a maximum of 356 μM, aliquoted, flash frozen in liquid N_2_ and stored at −80 °C until used.

### Molecular dynamics and pKa calculations

Molecular dynamics simulation and analysis of the wild-type LLO (PDB ID: 4CDB, ref. 17.) and Y406A mutant data were performed identically using GROMACS 4.6.4 or 4.6.5 software^47^. Default GROMACS settings were used when adding hydrogen atoms to starting structures. Both structures were solubilized in TIP3P water model^48^ and CHARM27 force field^49^ was used. Na^+^ and Cl^−^ ions were added to a final concentration of 300 mM. The system was energy minimized (steepest descent algorithm to F_max_ < 1000) and equilibrated for 0.1 ns at 310 K (V-rescale method^50^) and pressure of 1 bar (Parrinello-Rahman method^51^). The applied time step for all simulations was 2 fs. Periodic boundary conditions were applied in all three dimensions. Long-range electrostatics were evaluated by the Particle Mesh Ewald (PME) method^52^. Simulation time was 1 μs and 2 additional 200 ns simulations for both LLO and Y406A were performed. Radius of gyration was calculated with GROMACS tools. Protein structure images were prepared with PyMOL^53^. MD trajectory data was visualized with Visual Molecular Dynamics (VMD) software^54^. Data were plotted with Origin 8.1 (OriginLab, USA). A structural snapshot was extracted with GROMACS tools every 25 ns from the 1 μs long MD trajectory. These snapshots were uploaded to H ++ webserver version 3.1 and pH value of 7.4 was used for protonation prior to pK_a_ calculation^55^,^56^,^57^,^58^.

### Hemolytic assay

Bovine red blood cells (RBC) were washed 3–4 times in erythrocyte buffer. All erythrocyte buffers contained 140 mM NaCl besides 20 mM pH buffer (sodium acetate at pH 5, MES at pH 5.7, NaH_2_PO_4_/Na_2_HPO_4_ at pH 6, 6.5, 7, 7.4, 8 and 2-Amino-2-(hydroxymethyl)−1,3-propanediol (TRIS) at pH 8.5). Centrifugation at 800 × *g* at 21 °C for 4 minutes was followed by removal of supernatant and addition of fresh erythrocyte buffer to the pelleted RBC. After the last centrifugation the pellet was resuspended with the corresponding buffer to absorbance of ~1 at 630 nm (A_630_) as determined with the microplate reader Synergy MX (Biotek, USA). 100 μl serial dilutions (1:1) of either purified samples or cell lysates were prepared in 96 well clear microtiter plates and 100 μl of previously prepared RBC suspension was added to each well. A_630_ of all 96 wells was measured every 20 s for 20 min at 25 °C. V_max_ (OD/min) for each well was determined with Gen5 software (Biotek, USA) using linear regression with 3 data points. V_max_ data points were plotted against protein concentration (or sample volume in the case of cell lysates). To determine the concentration needed to achieve V_max_/2, curves were fitted with logistic function by using Origin 8.1 (OriginLab, USA) and the sigmoid midpoint (x_0_) was used.

### Calcein Release Experiments

All lipids in this study were purchased from Avanti Polar Lipids, USA, and used without further purification. 1-palmitoyl-2-oleoyl-*sn*-glycero-3-phosphocholine (POPC) and cholesterol (Chol) were dissolved in chloroform at molar ratio 1:1. Thin lipid film was generated using a rotavapor (Büchi, Switzerland) and left under high vacuum for 2–15 hours. Liposome buffer (10 mM MES (pH 5.7, 6, 6.5) or 10 mM NaH_2_PO_4_/Na_2_HPO_4_ (pH 7) or 10 mM 2-[4-(2-hydroxyethyl)piperazin-1-yl]ethanesulfonic acid (HEPES) (pH 7.5, 8), 150 mM NaCl, 1 mM ethylenediaminetetraacetic acid (EDTA), 50 mM calcein) with the pH corresponding to the starting conditions of each experiment was added to the lipid film. The sample was thoroughly vortexed together with 0.5 mm glass beads to resuspend the lipids. The MLV suspension was flash frozen and thawed at least three times. Large unilamellar vesicles (LUVs) were prepared by extrusion of MLVs with LiposoFast lipid extruder (Avestin, Canada) through polycyrbonate membranes with 100 nm pores. Excess calcein was removed from LUV suspension by gravity gel filtration on the Sephadex G-50 matrix (GE Healthcare, UK). Size and uniformity of the LUVs was checked with Dynamic Light Scattering (DLS). Concentration of POPC and Chol was enzymatically determined with Phospholipids C kit and Free Cholesterol E kit (Wako Diagnostics, USA), respectively. Permeabilization of calcein-loaded LUVs was assessed by using the Synergy MX microplate reader (Biotek, USA). Excitation wavelength was 485 nm and emission observed at 520 nm. Kinetic measurement started immediately after the protein sample was added. Final concentration of calcein-loaded LUVs was 20 μM in 200 μl. The release of calcein was followed for 700 s at which point detergent Triton X-100 was added to a final concentration of 2 mM to achieve full calcein release (100 % release). Negative control was addition of 2 mM Triton X-100 to LUV suspension without the LLO or Y406A. Data shown is one of three independent experiments.

### Giant Unilamellar Vesicles Imaging

Electroformation method was used for GUVs preparation. POPC and Chol 1:1 (mol:mol) were dissolved in chloroform. For membrane staining the rhodamine-DHPE (0.5 mol %) was added as fluorescent probe. Electroformation was carried out inside Vesicle Prep Pro device (Nanion Technologies, Germany) between two conductive ITO slides, where AC current was applied for 3 hours with increasing amplitude from 0 V to 4 V and a frequency of 10 Hz. The sucrose solution (290 mM sucrose, 1 mM MES, pH 5.5) was added to the dried lipid film on the conductive ITO slide. After the electroformation, the GUVs were sedimented with the glucose solution (290 mM glucose, 1 mM MES, pH 5.5), and the buffer was then exchanged to protein buffer (20 mM MES, 150 mM NaCl, pH 5.5). The osmolarity of the solutions was adjusted with the Osmomat 3000 osmometer (Gonotec GmbH, Germany). For the permeabilisation experiments, GUVs were mixed with protein buffer, fluorescent dextran (FD) of 70 kDa in size (final concentration of 1 mg/ml), proteins LLO or Y406A (final concentration of 100 nM) and incubated for 30 min at room temperature. As a negative control buffer alone was used instead of proteins. Images were recorded on DMI6000 CS inverted microscope with TCS SP5 laser scanning system (both Leica Microsystems, Germany) with a 40× oil-immersion objective (numerical aperture = 1.25). For excitation of FD70 and rhodamine the 488 nm and 543 nm line of lasers were used, and the fluorescence emission was detected from 502 to 529 nm and from 573 to 604 nm, respectively. Fluorescent signals were quantified in Fiji software^59^. Fluorescent intensities of FDs inside the vesicles were divided by background intensities outside the vesicles and represented as a percent of permeabilisation.

### Transmission Electron Microscopy

Multilamellar vesicles were prepared in 10 mM MES, 150 mM NaCl, pH 5.5 as described above. LLO and Y406A mutant were at 500 nM concentration and lipids were at 10 mM concentration. The vesicles suspension with bound proteins (2 μl) was applied to a Formvar-coated, carbon-stabilized grid and contrasted with 1% uranyl acetate (aqueous solution). Samples were imaged by CM 100 transmission electron microscope (Philips), equipped with Orius SC 200 camera (Gatan) and Digital Micrograph software.

### Liposome preparation for AFM imaging

Chol from ovine wool and POPC were used for liposome preparation. Briefly, lyophilized lipids in ratio 1:1 (mol:mol) were dissolved in organic solution chlorophorm:methanol 3:1 (vol:vol) to give a final concentration of 3 mM. Sample was transferred to a glass vial and dried under a flow of nitrogen. The lipid film was kept under reduced pressure overnight to ensure the absence of organic solvent traces. Then, the lipid film was hydrated with Milli-Q water to give a final lipid concentration of 500 μM, subjecting the vials to 5 cycles of agitation of 1 min, and heating to ~70 °C, well above the transition temperature of the lipid mixture studied herein. The obtained MLVs were sonicated for 40 minutes in order to obtain LUVs. LUV suspensions were stored at 4 °C and used during maximal 10 days. During all the preparation processes, samples were protected from light to avoid unspecific oxidation.

### Supported bilayer preparation

Supported Lipid Bilayers (SLBs) were prepared by fusion of LUVs on the mica support, adapted from^60^. To form the SLBs, 2 μL of LUVs were deposited on 1.5 mm^2^ freshly cleaved mica surface, which was glued with epoxy to the quartz sample stage. After incubating the sample for 30–40 minutes in a humid chamber it was gently rinsed with Milli-Q water and never left to dry^21^,^61^.

### HS-AFM imaging

High-speed atomic force microscopy (HS-AFM, RIBM, Japan) movies were acquired using 8 μm-long cantilever (USC-1.2, Nanoworld, Neuchatel, Switzerland) with nominal spring constant *k* = 0.15 N/m and resonance frequency *f* = 0.6 MHz in solution. Both the cantilever and the rinsed mica surface with incubated bilayer were placed into a ~120 μL imaging buffer chamber. HS-AFM was operated in oscillating mode. Small oscillation free and set point amplitude of about 1 nm and 0.9 nm, respectively, were used, to achieve minimum tip-sample interaction^62^. LLO and Y406A mutant were added to a final concentration of 500 nM after identification of the membrane patches on the mica surface. HS-AFM measurements and observations of LLO and Y406A mutant were performed at room temperature. Buffer was 20 mM MES, pH5.6, 100 mM NaCl, 5 mM MgCl_2_. HS-AFM image and data processing were performed using ImageJ software^63^ with dedicated plugins developed for HS-AFM^64^. All further analysis, i.e. histogram distributions were analyzed with software Matlab (The Mathworks, USA) and Origin (OriginLab, USA).

### Electrical measurements

For electrical measurements in planar lipid bilayers an integrated chip-based recording setup Orbit mini and EDR2 software (Nanion Technologies, Germany) were used. Recordings were obtained in parallel with multi-electrode-cavity-array (MECA) chips (Ionera Technologies, Germany). In all experiments bilayers were obtained from a mixture of 1,2-diphytanoyl-*sn*-glycero-3-phospho-choline (DPhPC), POPC and Chol in ratio 1:1:2 (mol:mol:mol) dissolved in octane (final concentration of lipids 5 mg/ml). The electrolyte solution was 1 M KCl, 20 mM MES, pH 5.5. LLO or Y406A was added to the *cis* side of the bilayer to a final concentration of ~4 μM. A voltage of +40 mV was applied to facilitate pore insertion. Sampling rate was 20 kHz. Current traces were analyzed and standard deviation/noise (pA) was measured at 5 s interval using Clampfit (10.6).

### Calcein Release Activation Assay

Calcein-loaded LUVs were prepared and free calcein was removed as described above. pH inside LUVs was matched to that of the pH at the start of the experiment (pH 6.5 or pH 8). Final concentration of LUVs was 20 μM and of proteins was 0.1 μM in a cuvette with the 3 ml final volume. Baseline fluorescence was measured for 300 s when Y406A mutant was added. Controlled pH variation was achieved with the addition of 16 μl of 4 % HCl (Merck, Germany) directly into the sample during measurement that started at pH 8 at approximately 400 s. This changed the pH to ~6.5. This change in pH did not release any calcein from the vesicles in the absence of Y406A. Fluorescence was then followed for 1700–1800 s when Triton X-100 was added to a final concentration of 2 mM to achieve full release of calcein. All data from fluorescence experiments was analyzed and drawn with Origin 8.1 (OriginLab, USA). Data shown is one of three independent experiments.

### Surface Plasmon Resonance Activation Assay

SPR activation assay was performed on Biacore X (GE Healthcare, UK) at 25 °C and L1 sensor chips. SPR vesicle buffer (20 mM NaH_2_PO_4_/Na_2_HPO_4_, 140 mM NaCl, pH 5.7 or 7.4) with added 50 mM calcein was used to prepare calcein-loaded LUVs as described above. Calcein-loaded LUVs were loaded to L1 chip (at a flow-rate 2 μl/min for 600 s) and equilibrated in SPR buffer (20 mM NaH_2_PO_4_/Na_2_HPO_4_, 150 mM NaCl, 1 mM EDTA, pH 8) at 60 μl/min for 600 s. In this step free calcein was removed from the system. Equilibration at pH 8 was followed by a wash with SPR buffer pH 5.7 (30 μl/min, 90 s) and this was the first collected fraction (marked as buffer in Fig. 5b and c). Next an injection of 250 nM LLO or Y406A (30 μl/min, 90 s, association phase) followed by a wash at 30 μl/min and 90 s (dissociation phase). The cell was further washed with the SPR buffer (30 μl/min, 90 s, marked as Background). This was followed by an injection of an SPR buffer at pH 5.7 (30 μl/min, 90 s, marked as Buffer). Finally the chip was washed with 40 mM octyl-glucoside detergent (OG) to release all remaining material from the surface of the sensor chip. (30 μl/min, 270 s, termed 40 mM OG). All SPR data were processed in BIAevaluation v3.2 (GE Healthcare, UK) software. Experiments were repeated independently three times.

### Cell Culture Assay

Caco-2 cells were grown at 37 °C, 5 % CO_2_ in Minimum essential medium eagle (MEM) supplemented with 10 % (vol %) fetal bovine serum (FBS), 1 % L-glutamine and 1 % non-essential amino acids. For experiments cells were seeded on 96-well microtiter plates or chamber slides in Dulbecco′s Modified Eagle′s Medium (DMEM) supplemented with 1 % L-glutamine, 1 % antibiotic-antimycotic (Thermo Fisher Scientific, USA). For fluorescent dye uptake measurement, cells were seeded in a microtiter plate and were washed with DMEM pH 7.4 and Y406A was added to a final concentration of 0.5 μM followed by 5 minutes of incubation at room temperature. Cells were washed with DMEM pH 7.4 to remove any unbound Y406A. In the next step half of the wells were washed twice with DMEM pH 7.4 and the other half first with DMEM adjusted to pH 6. After washing, SYTOX Green (Invitrogen, USA) was added in identical medium to a final concentration of 15 μM. SYTOX Green fluorescence was measured after 30 min at 520 nm with FLUOstar Galaxy microplate reader (BMG, Germany). In a positive control 0.9 % (vol/vol) Triton X-100 was added to the cells and negative control were cells washed with DMEM. For confocal microscopy the Caco-2 cells on chamber slides were treated and stained as described for microplate measurements. DMI6000 CS inverted microscope with TCS SP5 laser scanning system (both Leica Microsystems, Germany) was used for imaging. For sequential excitation, a 50-mW 405 nm diode and a 476 nm line of a 25 mW argon laser were used. The fluorescence emission was detected between 500 and 529 nm. Images were analysed by using Leica Application Suite Advanced Fluorescence (LAS AF) Lite software version 2.5.1.

## Additional Information

**How to cite this article:** Kisovec, M. *et al*. Engineering a pH responsive pore forming protein. *Sci. Rep.* **7**, 42231; doi: 10.1038/srep42231 (2017).

**Publisher's note:** Springer Nature remains neutral with regard to jurisdictional claims in published maps and institutional affiliations.

## Supplementary Material

Supplementary Information
