# Supplementary material for: Engineering a pH responsive pore forming protein
Source: Sci Rep. 2017 Feb 8;7:42231. doi: 10.1038/srep42231 (PMC5296754; doi:10.1038/srep42231)
Supplement: Supplementary Information [file srep42231-s1.pdf]

Supporting Information

Engineering a pH responsive pore forming protein

Matic Kisovec[1], Saša Rezelj[1], Primož Knap[1,2], Miša Mojca Cajnko[1], Simon Caserman[1], Ajda Flašker[1], Nada Žnidaršič[3], Matej Repič[4], Janez Mavri[4], Yi Ruan[5], Simon Scheuring[5], Marjetka Podobnik[1], Gregor Anderluh[1,*]

[1] Department of Molecular Biology and Nanobiotechnology, National Institute of Chemistry, Hajdrihova 19, 1000 Ljubljana, Slovenia

[2] Present address: Istituto di Biofisica, Consiglio Nazionale delle Ricerche & Fondazione Bruno Kessler, via alla Cascata 56/C, 38123 Trento, Italy

[3] Department of Biology, Biotechnical Faculty, University of Ljubljana, Jamnikarjeva 101, 1000 Ljubljana, Slovenia

[4] Department of Computational Biochemistry and Drug Design, National Institute of Chemistry, Hajdrihova 19, 1000 Ljubljana, Slovenia

[5] U1006 Institut National de la Santé et de la Recherche Médicale (INSERM), Université Aix-Marseille, Parc Scientifique et Technologique de Luminy, 163 avenue de Luminy, 13009, Marseille, France

Address correspondence to gregor.anderluh@ki.si

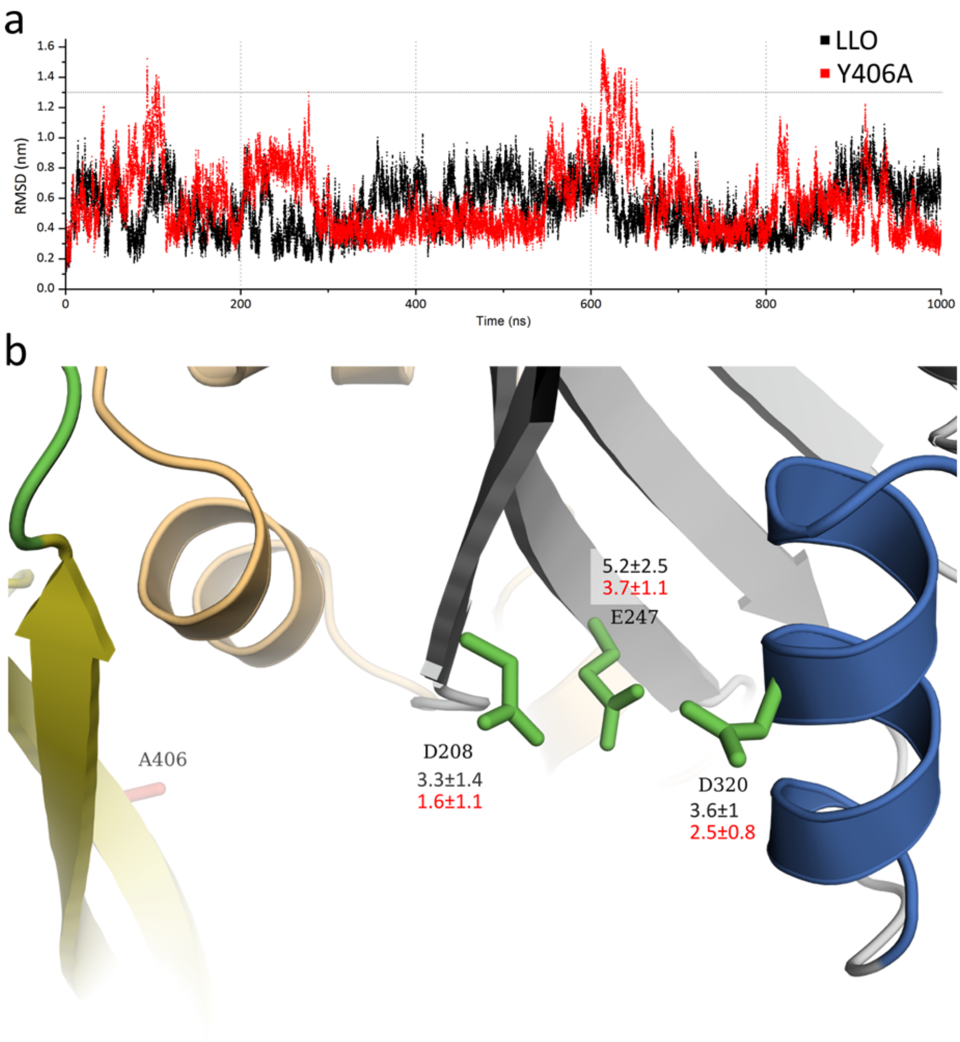

Supplementary Figure S1. Molecular dynamics simulation RMSD and calculated pKa values. (a) Root-mean-square deviations of entire (D1-D4) LLO and Y406A from 1 μs long MD simulations. Compared to Figure 2a (R_g of D1-D3) the RMSD fluctuates more because the D4 is very flexible. Values above 1.3 nm (horizontal dashed line) represent the "opened state". (**b**) Close-up of Y406A mutant structure. The MD snapshot is an example of the "opened state". pH sensor residues D208, E247 and D320 (green sticks) have their pKa values indicated. Each residue name is accompanied with pKa ± SD of the wild-type LLO (black) and pKa ± SD of Y406A (red), where SD stands for standard deviation. Please note that the reference pK_a values of the residues in the aqueous solution are 10.7 for Lys, 3.7 for Asp, 4.2 for Glu and 10.1 for Tyr. Residue Ala406 is shown in red stick. Domain colours are the same as in Figure 1.

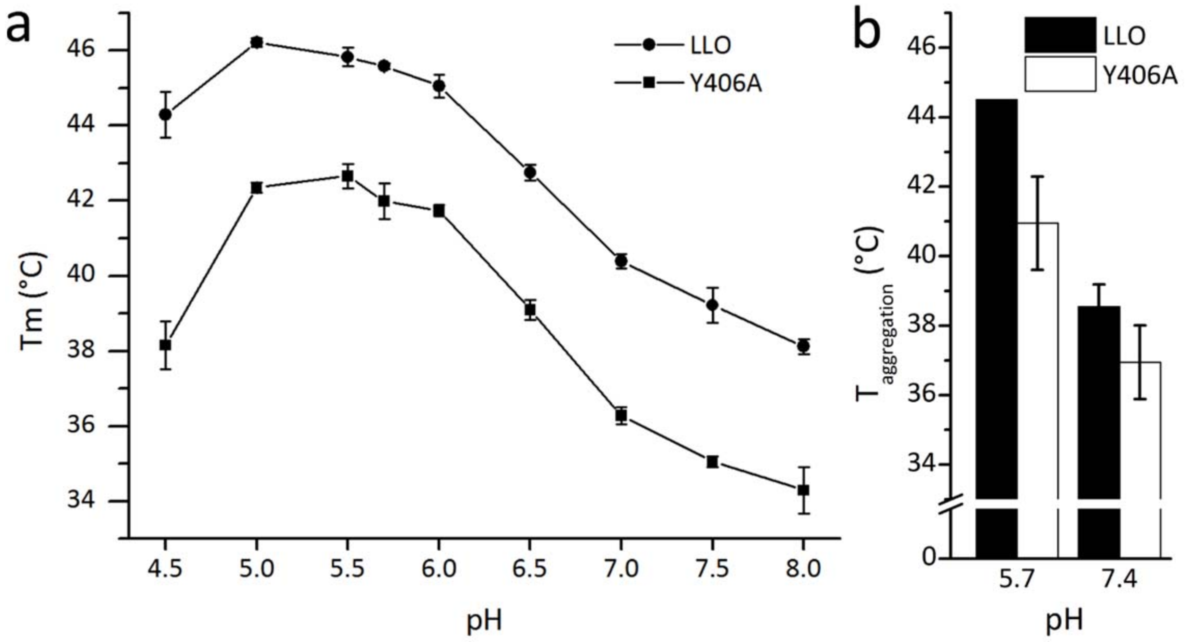

Supplementary Figure S2. Thermal stability of LLO and Y406A mutant. (**a**) Melting temperatures (T_m) at different pH values as determined with DSF. The pH dependent stability of Y406A is negatively offset for about 4 °C compared to LLO. Average ± SD, n=3. (**b**) Temperature of LLO and Y406A aggregation as determined spectrophotometrically. Average ± SD; n=2.

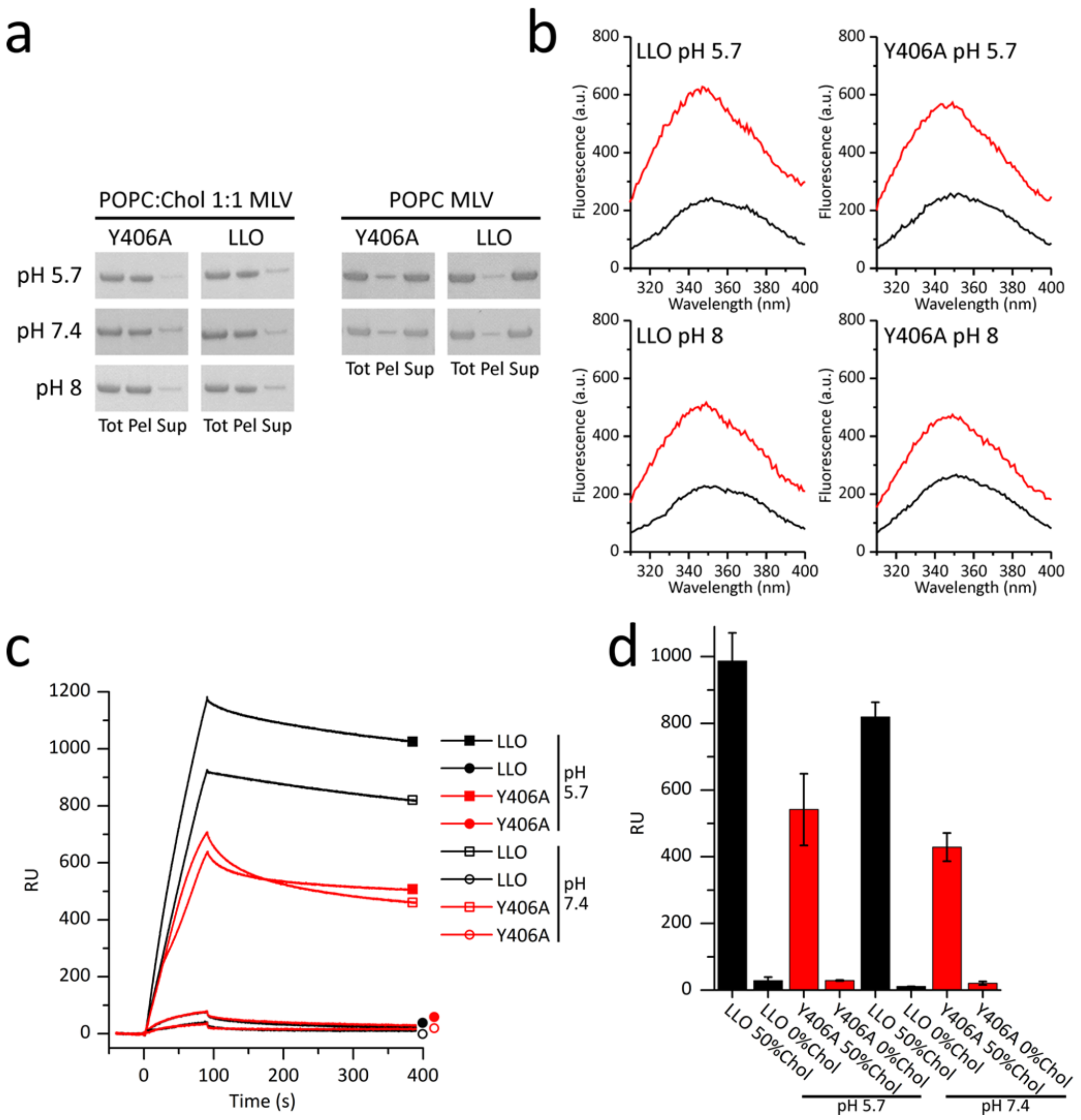

Supplementary Figure S3. Binding of LLO and Y406A to lipid membranes. Both proteins bind POPC:Chol 1:1 (mol:mol) membranes in the pH range from pH 5.7 to pH 8. (**a**) SDS PAGE of proteins after binding to MLVs. Tot – total added protein, Pel – pelleted fraction, Sup – supernatant fraction. (**b**) Tryptophan fluorescence change after the addition of MLVs at two different pH values. (**c**) SPR sensogram of LLO (black) and Y406A mutant (red) binding to lipid-coated SPR surface at pH 5.7 (empty symbols) or 7.4 (full symbols). Lipid coating included POPC with either 50 mol % of cholesterol (square) or 0 mol % cholesterol (circle). (**d**) Binding of LLO (black) and Y406A (red) mutant to lipid-coated SPR surface at pH 5.7 and 7.4. Histogram is derived from sensorgram (time = 300 s) such as shown in panel d. Error bars represent ±SD, n=3.

MATERIALS AND METHODS

Temperature and pH stability of monomers

Temperature dependent protein aggregation was determined spectrophotometrically with absorbance at 600 nm. LLO WT and LLO Y406A were diluted to 4 µM concentration in 20 mM NaH_2PO_4/Na_2HPO_4, 140 mM NaCl pH 5.7 or pH 7.4. Sample volume was 1300 µl and the sample was heated from 15°C to 70°C at a rate 1 °C/min with constant stirring. Data was plotted in Origin 8.1 (OriginLab, USA) and T_m determined by fitting a Boltzmann function.

pH dependent thermal stability of LLO and Y406A was determined with Differential Scanning Fluorometry (DSF aka. Thermal Shift Assay, TSA). A series of buffers with a pH range from pH 4.5 to 8 (20mM CH_3COON, 150 mM NaCl) was used. Sypro Orange (Thermo Fisher Scientific, USA) final dilution was 1:2500 and final protein concentration was 1.8 µM. Reaction volume was 50µl. Fluorescence was followed with LightCycler 480 (Roche, Switzerland) from 25 ºC to 95 ºC at a gradient ~1ºC/min. Boltzmann function in Origin 8.1 (OriginLab, USA) was used to determine the T_m in each well. Each condition was duplicated twice on a single 96-well plate and experiment was repeated independently three times.

Binding to multilammelar vesicle (MLV) membranes

LLO or Y406A mutant was added to the MLV suspension 1:3000 (mol:mol) to a final volume of 20 µl and incubated for 40-60 minutes at 21 ºC. The suspension was centrifuged at 16 000 *xg*, 20°C for 15 minutes and the supernatant was carefully removed. The pelleted MLV fraction was resuspended in 50 µl of the MLV buffer with the corresponding pH and centrifuged again. The second supernatant fraction and the second pelleted fraction were analyzed with SDS-PAGE.

Tryptophan fluorescence

Protein was diluted in 10 mM MES, 150 mM NaCl, 1 mM EDTA, pH 5.7 or 10 mM HEPES, 150 mM NaCl, 1 mM EDTA pH 8 buffer to a final concentration of 0.89 µM and a final volume of 1 ml. Tryptophan fluorescence spectra were measured with fluorometer LS 55 (PerkinElmer, USA) in a glass quartz cuvette with constant mixing at 21°C. Excitation wavelength was 295 nm (slit 5 nm) and 300 – 400 nm (slit 3 nm) emission spectra was recorded. Each protein sample was measured without and with 88µM POPC:Chol (1:1 mol:mol) LUVs at both pH values. LUVs were prepared as for calcein release experiments only without the calcein in the buffer. Data shown is one of three independent experiments.

Surface plasmon resonance binding assay

SPR experiments were performed by using Biacore X (GE Healthcare, UK) at 25 ºC and L1 sensor chips. SPR vesicle buffer (20 mM NaH_2PO_4/Na_2HPO_4, 300 mM NaCl, pH 5.7 or 7.4) was used to prepare LUVs as described in calcein release paragraph. LUVs were loaded to L1 chip (at a flow-rate 1 µl/min for 600 s), the chip washed with 100 mM NaOH (flow-rate 30 µl/min for 120 s), washed with 0.1 mg/ml BSA (flow-rate 30 µl/min for 60 s), equilibrated with SPR vesicle buffer (flow-rate 30 µl/min for 390 s). Next an injection of 30 nM LLO or Y406A (30 µl/min, 90s) followed by a wash at 30 µl/min and 300s. The cell was further washed with the 100 mM NaOH (30 µl/min, 60 s). All SPR data was processed in BIA evaluation v3.2 (GE Healthcare, UK) software. Experiments were repeated independently three times.
